# Supplementary material for: Priorities for Research into the Impact of Canine Surgical Sterilisation Programmes for Free-Roaming Dogs: An International Priority Setting Partnership
Source: Animals (Basel). 2021 Jul 30;11(8):2250. doi: 10.3390/ani11082250 (PMC8388514; doi:10.3390/ani11082250)
Supplement: Supplementary file 1 [file animals-11-02250-s001.zip › File S1 JLA Sterilisation PSP Protocol.pdf]

File S1 Impact of Canine Surgical Sterilisation Programmes: Priority Setting Partnership Protocol

[20/01/19]

1. Purpose of the PSP and background

The purpose of this protocol is to set out the aims and objectives of the Impact of Surgical Sterilisation Priority Setting Partnership (PSP).

A priority setting partnership (PSP) is traditionally used in human medicine to bring patients, carers and clinicians together to identify and prioritise uncertainties, or 'unanswered questions' (questions that have not already been answered by existing research) regarding a certain health problem. The aim is to involve people who are directly affected by a problem, but who may not usually have a chance to influence the research agenda.

This PSP will adapt the methodology to bring together people who are directly involved in canine surgical sterilisation programs e.g. as program managers, vets or assistants, to identify and prioritise what they think are the most important uncertainties regarding the impact of these programs. It is hoped that this will identify directions for future research that are directly applicable to the organisations who are carrying out these programs.

A systematic review recently undertaken showed that evidence of the impact of canine sterilisation programs, particularly their role within a rabies control program, is limited. Attempts to control rabies are often aligned with surgical sterilisation programs which have different, but potentially synergistic, aims of stabilising/reducing dog populations and improving the health and welfare of free-roaming dogs. A lack of monitoring and evaluation of outcomes in many of these programs makes it difficult to optimise their effectiveness.

2. Aims and objectives of the Impact of Canine Surgical Sterilisation Programs PSP

The aim of this PSP is to identify and prioritise unanswered questions about the impact of sterilisation programs on a population of free-roaming dogs and their role within a rabies control program.

The objectives are to:

- work with individuals and organisations (e.g. non-governmental organisations (NGOs), international government organisations (IGOs), governmental departments) who have direct experience of working on canine surgical sterilisation programs to identify uncertainties about the impact of sterilisation programs on a population of free-roaming dogs.
- work with individuals and organisations (as above) who have direct experience of working on canine surgical sterilisation and/or canine rabies control programs to identify uncertainties about the impact of sterilisation programs on rabies control.
- agree by consensus a prioritised list of those uncertainties
- publicise the results of the PSP and process
- take the results to research commissioning bodies to be considered for funding.

3. The Steering Group

The PSP will be led and managed by the following:
Abi Collinson, PhD student at the Centre for Evidence-based Veterinary Medicine (CEVM), University of Nottingham

Advisors will include Jenny Stavisky and Malcolm Bennett (University of Nottingham) plus representatives from NGOs and governmental organisations who work in dog population management. The steering group will have a wide range of experiences from different geographical and institutional settings to reflect the differences between free roaming dog populations, and how they are managed globally.

The Partnership and the priority setting process will be supported and guided by: Rachel Dean, who has previous experience of conducting an adapted JLA PSP in the field of Veterinary science.

4. Participants

Organisations and individuals will be invited to be involved with the PSP by answering and disseminating the initial survey to gather questions of practical importance, and subsequent survey to rank those questions in order of importance. They will also be invited to register their interest to participate in a final workshop to decide on the final 'top 10' prioritised questions:

Inclusion criteria

- People (e.g. program managers, vets, vet technicians, animal health assistants, government workers) who have direct experience of working on canine surgical sterilisation programs or canine rabies control programs in any country

Exclusion criteria
There are no organisations that are judged to have conflicts of interest.

In traditional JLA's researchers cannot participate in the prioritisation exercise. This is to ensure that the final prioritised research questions are those agreed by patients, carers and clinicians only, in line with the JLA's mission. In this adapted method we have decided to also exclude researchers from the process, in order to let people working in the field have the opportunity to influence the research agenda.

5. Methods

This section describes a schedule of proposed stages through which the PSP aims to fulfil its objectives. The process is iterative and dependent on the active participation and contribution of different groups. The methods adopted in any stage will be agreed through consultation between the Steering Group members, guided by the PSP's aims and objectives

Step 1: Identification and invitation of potential participants
Potential participants will be identified through a process of peer knowledge, contacts made from previous work and from the systematic review process. Potential participants will be contacted and informed of the establishment and aims of the PSP and how to become involved.

Step 2: Awareness raising

The initial awareness raising will be in the form of an email with a link to the initial survey, and will have several key objectives:

- to present the proposed plan for the PSP
- to establish principles upon which an open, inclusive and transparent mechanism can be based for contributing to, reporting and recording the work and progress of the PSP.

Step 3: Identifying treatment uncertainties

An online questionnaire will be developed to identify questions and uncertainties about:
1) The impact of sterilisation programs on a population of free roaming dogs
2) The impact of sterilisation in a canine rabies control program

The steering group will discuss the best method for distributing the initial survey. It is anticipated that the initial survey will be open for 4 weeks.

Existing sources of information about uncertainties for these programs will be searched. This will include research recommendations in evidence syntheses, and other evidence in peer-reviewed literature, reports and guidelines.

Step 4: Refining questions and uncertainties

The questionnaire will produce “raw” unanswered questions about the impact of program components, and improving the impact of programs. These raw questions will be assembled, categorised and refined into “collated indicative questions” which are clear, addressable by research and understandable to all. Similar questions will be combined where appropriate.

If uncertainties are expressed that can be resolved with reference to existing research evidence and are "unknown knowns", and not uncertainties, - this evidence will be highlighted as it suggests that information is not being communicated effectively to those who need it.

Uncertainties which are not adequately addressed by previous research will be collated for interim (if applicable) and final prioritisation.

Step 5: Prioritisation – interim and final stages

The aim of the final stage of the priority setting process is to prioritise through consensus the identified questions.

The interim stage, to proceed from the full long list of questions to a shorter list (<30), will be carried out online by participants who completed the questionnaire and agreed to participate in future research. They will be asked to choose and rank their top 10 most important uncertainties.

The final stage, to prioritise the short listed uncertainties and agree a top 10, will be conducted using an online or in-person (or a combination of both) meeting, using group discussions and plenary sessions.

The methods used for this prioritisation process will be determined by consultation with the steering group during the process. Methods which have been identified as potentially useful in this process include: adapted Delphi techniques; expert panels or nominal group techniques; consensus development conference; electronic nominal group and online voting; interactive research agenda setting and focus groups.

6. Dissemination of findings and research

Findings and research
It is anticipated that the findings of the PSP will be reported to funding and research agenda setting organisations. The prioritised uncertainties will be developed into research questions, and the research needs of those unanswered questions will be established.

Publicity
The results will be communicated to all interested participants, as well as to potential funders. A lay summary will be disseminated to organisations working in the field. The findings will form part of a PhD thesis and will be published as an academic paper in an open access peer-reviewed journal.
