# Supplementary material for: Priorities for Research into the Impact of Canine Surgical Sterilisation Programmes for Free-Roaming Dogs: An International Priority Setting Partnership
Source: Animals (Basel). 2021 Jul 30;11(8):2250. doi: 10.3390/ani11082250 (PMC8388514; doi:10.3390/ani11082250)
Supplement: Supplementary file 1 [file animals-11-02250-s001.zip › File S2 Initial Scoping Survey.pdf]

Impact of Canine Surgical Sterilisation Programs: Priority Setting Partnership Survey

---

Background

We would like people who have direct, first-hand experience of working on dog population management and/or canine rabies control programs to let us know what questions they have about the impact of their programs.

Canine surgical sterilisation programs are a common intervention used to manage free-roaming dog populations in many parts of the world. The aims of such programs are varied but may include stabilising/reducing the population, improving the health and welfare of free-roaming dogs and reducing problems associated with the population e.g dog-bites and rabies transmission.

A systematic review recently undertaken has shown that there is a lack of studies which evaluate the impact of sterilisation programs, particularly their role within a rabies control program.

A priority setting partnership is a method for identifying future research priorities. There are three stages - an initial survey to identify what questions people have, an interim prioritisation where the questions are ranked in order of importance and a final prioritisation process which results in an agreed list of top 10 questions for researchers to answer.

If you want to read more about this Priority Setting Partnership (PSP) you can visit our website by clicking on this link:

www.nottingham.ac.uk/cevm/impactcaninesurvey

Data Protection Notice

How will my information be used?

The survey can be completed anonymously. There is no need to provide contact details unless you would like to take part in the next stages of the project or hear about the survey results. If you do provide contact details they will be kept separate from your survey responses so that you will not be personally identifiable in the results. We will ask some questions about your role, organisation and where you live and work; this information will not be used for any purpose other than that stated, and will not be shared outside of the study. We will keep the information that you give us secure in accordance with the Data Protection Act 1998 and with GDPR Legislation 2018.

For information about the University of Nottingham's obligations with respect to your data, who you can get in touch with and your rights as a data subject, please visit: www.nottingham.ac.uk/utilities/privacy/privacy.aspx.

The study is being led by the Centre for Evidence-based Veterinary Medicine (CEVM) and has received ethical approval from the University of Nottingham School of Veterinary Medicine and Science.

For further information or if you have any concerns or complaints please contact us:

Abi Collinson BVetMed MRCVS: abigail.collinson@nottingham.ac.uk

Jenny Stavisky BVMS PhD MRCVS: jenny.stavisky@nottingham.ac.uk

Please tick the box below if you are happy to participate in this survey ✱ *Required*

○ Yes, I have read and understood the information above and consent to participate

What do you need to do?

Think about your experiences and understanding of surgical sterilisation programs and combined sterilisation and rabies vaccination programs. We would like you to tell us what questions you have that could make a difference to how you might plan, implement, and monitor your programs. They can be about anything that you feel is important.

The survey is completed anonymously. It has 3 sections, not all of the questions may be relevant to you and you do not have to answer all of the sections. The survey can be saved part way through.

Section 1: is for information about yourself. This is so that we can ensure that a wide range of people have taken part

Section 2: is for you to tell us what your questions are about the impact of surgical sterilisation programs on a population of free-roaming dogs.

Section 3: is for you to tell us what your questions are about the impact of surgical sterilisation when used alongside vaccination in a canine rabies control program.

This survey will close for responses on 7th April 2019

Thank you for taking the time to complete this survey.

Definitions

Definitions for the purposes of this survey:

Free-roaming dogs: Dogs that are not confined. They may be owned, semi-owned or unowned.

Surgical sterilisation: surgical removal of the reproductive organs. Also known as spaying or neutering, animal birth control (ABC), catch-neuter-release (CNR).

Surgical sterilisation program: any type of program in which large numbers of free-roaming dogs are sterilised. This could involve owners bringing the dogs, dogs being caught or a combination of both, and could be conducted from a static site e.g. clinic or shelter or at temporary mobile field sites.

Do you have direct experience of working on dog population management programs that have included surgical sterilisation and/or rabies vaccination? *

Required

Section 1: About you and your work

It is important to know a little bit about you and the type of work you are involved in to make sure we get responses from a wide range of people and so we can understand the research questions that concern different groups. The questions in this section will allow us to do that.

Which of the following best describes you? *Tick all that apply* ✱ *Required*

- ☐ Veterinarian
- ☐ Veterinary nurse/technician
- ☐ Program manager
- ☐ Government worker (please specify department/role)
- ☐ Community animal health worker/Animal health technician
- ☐ Academic/Researcher
- ☐ Other (please specify)

If you selected 'government worker' or other' - please specify

Which of the following best describes the organisation that you work for currently, or have worked for? *Tick all that apply* ✱ *Required*

- ☐ Non-governmental organisation (NGO)
- ☐ International governmental organisation (IGO)
- ☐ Governmental organisation (in one country)
- ☐ Academic/research institution

- [ ] I am an independent consultant
- [ ] I have done voluntary work (please specify)
- [ ] Other (please specify)

If you selected 'other' - please specify:

What is the name of your organisation (optional)

Which of the following best describes the **main** objective of your organisation?
**Public health (human) refers to organisations working to protect and improve human health and well-being. Public health (veterinary) refers to organisations working to protect and improve human health and well-being specifically through veterinary work.*
✱ *Required*

- [ ] Animal health
- [ ] Human health/ Public health (human)
- [ ] Public health (veterinary)
- [ ] Animal welfare
- [ ] Community engagement
- [ ] Education/training
- [ ] Other (please specify)
- [ ] Unsure/Don't know

If you selected other - please specify:

Which other objectives does your organisation have? Tick all that apply

- ☐ Animal health
- ☐ Human health/Public health (human)
- ☐ Public health (veterinary)
- ☐ Animal welfare
- ☐ Community engagement
- ☐ Education/training
- ☐ None of the above
- ☐ Other (please specify)

If you selected other - please specify:

Which country do you currently **live** in? ✱ *Required*

Please list the countries in which you **work**, or have worked, with free-roaming dogs ✱ *Required*

Section 2: What questions do you have about the impact of surgical sterilisation programs on a population of free-roaming dogs?

Please write down any questions that you have about what impact surgical sterilisation programs have on a population of free-roaming dogs. This may include questions about how you plan and carry out your programs in order to acheive certain impacts. Do not worry if you think your questions may already have been answered. Just think about what interests you and write down anything that you would like to know the answer to.

These may include effects on **dog longevity, health, welfare, behaviour, population size, structure** and **turnover** (births and deaths in the population) or effects on the **local community** such as **changes in attitudes and behaviour** towards free-roaming dogs and/or the program.

An example would be "do sterilised free-roaming dogs live longer?" or "does a sterilisation program reduce the size of the dog population?" (*If either of these examples were going to be one of your questions then please still write them in the box below).* You can write as many questions as you want. Please write each new question on a new line.

Section 3: What questions do you have about using surgical sterilisation as part of a canine rabies control program?

The impact of surgical sterilisation in canine rabies control is widely debated. If you do not work in an area where rabies is endemic, or have not been involved in combined surgical sterilisation and rabies vaccination programs then this section may not apply to you. Thank you for answering the previous sections.

Please write down any questions you have about the impact of using combined surgical sterilisation and rabies vaccination as part of a canine rabies control program. Think about how and why you use sterilisation alongside vaccination (or why you don't use sterilisation if you are involved in vaccination only programs) and your reasons for doing so. *An example would be: "if I am carrying out sterilisation alongside rabies vaccination do I still need to vaccinate as many dogs?" or "if I am conducting a catch-neuter-release program, do I need to revaccinate the dogs every year" or "are owners more likely to bring sterilised dogs to be vaccinated against rabies?"(If any of these examples were going to be one of your questions then please still write them in the box below).* Please write each new question on a new line.

Would you like to stay in touch?

Once the survey has closed the submitted questions will be grouped into research questions. Those who are interested will then be asked to rank those questions in order of importance.

If you would like a summary of the results and/or are happy to be contacted to be involved in the next stages of the project then please complete the following information:

I would like: (please tick one or both of the options)

- ☐ A summary of the results
- ☐ To continue to be involved in the project

Email address:

First name:

Last name:

Final page

Thank you for taking the time to complete this survey. Your responses will help us to identify directions for future research on canine sterilisation and rabies control programs that are directly applicable to organisations who are carrying out these programs. Please visit our project page to learn more or for updates on the project.

www.nottingham.ac.uk/cevm/impactcaninesurvey

If you have any questions please email Abi Collinson at: abigail.collinson@nottingham.ac.uk or Jenny Stavisky at: jenny.stavisky@nottingham.ac.uk

---

Key for selection options

2 - Do you have direct experience of working on dog population management programs that have included surgical sterilisation and/or rabies vaccination?

Yes
No
