# Supplementary material for: Priorities for Research into the Impact of Canine Surgical Sterilisation Programmes for Free-Roaming Dogs: An International Priority Setting Partnership
Source: Animals (Basel). 2021 Jul 30;11(8):2250. doi: 10.3390/ani11082250 (PMC8388514; doi:10.3390/ani11082250)
Supplement: Supplementary file 1 [file animals-11-02250-s001.zip › File S3 Full demographic data.pdf]

File S3 Demographic data of all survey respondents

1. Initial survey

1.1 Countries of residence

- Countries/regions of residence (as determined by the United Nations geoscheme which divides countries of the world into regional and sub-regional groups):

Region	Sub-region	Countries	No.
Africa		Ethiopia<br>Ghana<br>Kenya<br>Liberia<br>Malawi<br>Namibia<br>Nigeria<br>South Africa<br>South Sudan<br>Swaziland<br>Tanzania<br>Uganda<br><br>TOTAL - 12	1<br>1<br>1<br>1<br>2<br>2<br>2<br>1<br>1<br>1<br>2<br>1<br><br>16
Americas	North America	Canada<br>USA<br><br>TOTAL: 2	2<br>13<br><br>15
	Latin America and the Caribbean	Bahamas<br>Belize<br>Chile<br>Costa Rica<br>Ecuador<br>El Salvador<br>Grenada<br>Guatemala<br>Haiti<br>Mexico<br>Nicaragua<br>Panama<br><br>TOTAL: 12	1<br>1<br>2<br>2<br>1<br>1<br>1<br>1<br>1<br>3<br>1<br>1<br><br>16
Asia	South Asia<br><br><br><br><br><br>South Eastern Asia	Afghanistan<br>Bangladesh<br>India<br>Nepal<br>Pakistan<br><br>Indonesia<br>Malaysia<br>Philippines<br>Thailand<br>Vietnam	1<br>1<br>12<br>2<br>4<br><br>1<br>4<br>1<br>2<br>1

	Western Asia	Oman<br>Iraq<br><br>TOTAL: 12	1<br>1<br><br>31
Oceania		Australia<br>New Zealand<br><br>TOTAL: 2	9<br>1<br><br>10
Europe		Bulgaria<br>Bosnia and Herzegovina<br>Denmark<br>Ireland<br>Netherlands<br>Portugal<br>United Kingdom<br><br>TOTAL: 7	1<br>1<br>1<br>1<br>1<br>1<br>16<br><br>22

1.2 Countries worked in

Region	Sub-region	Country	Number
Africa	Northern Africa:<br><br><br><br><br>Sub-Saharan Africa:	Egypt<br>Morocco<br>Tunisia<br><br>Botswana<br>Chad<br>Ethiopia<br>Gambia<br>Ghana<br>Kenya<br>Liberia<br>Malawi<br>Mauritius<br>Mozambique<br>Namibia<br>Nigeria<br>Rwanda<br>Sierra Leone<br>South Africa<br>South Sudan<br>Swaziland<br>Tanzania<br>Uganda<br><br>TOTAL: 22	2<br>2<br>1<br><br>1<br>1<br>1<br>1<br>3<br>5<br>1<br>6<br>2<br>1<br>2<br>3<br>1<br>1<br>3<br>1<br>1<br>7<br>2<br><br>48
Americas	North America:<br><br>Latin America: and the Caribbean	Canada<br>USA<br><br>Argentina<br>Bahamas<br>Belize<br>Brazil<br>Bolivia	6<br>6<br><br>2<br>1<br>5<br>2<br>1

		Chile	2
		Colombia	5
		Costa Rica	4
		Dominican Republic	8
		Ecuador	11
		El Salvador	2
		Guatemala	11
		Guyana	1
		Haiti	2
		Honduras	7
		Jamaica	2
		Mexico	10
		Nicaragua	11
		Panama	5
		Paraguay	3
		Peru	9
		St Kitts and Nevis	1
		St Lucia	1
		Suriname	1
		Uruguay	2
		TOTAL:27	109
Asia	Eastern Asia	China	1
		South Korea	1
	South Eastern Asia:	Cambodia	8
		Indonesia	5
		Laos	4
		Malaysia	5
		Philippines	2
		Thailand	17
		Vietnam	2
	Southern Asia:	Afghanistan	1
		Bangladesh	2
		Bhutan	1
		India	35
		Iran (Islamic republic of)	1
		Nepal	18
		Pakistan	4
		Sri Lanka	9
	Western Asia:	Armenia	1
		Iraq	1
		Jordan	1
		Oman	1
		Qatar	1
		Syria	1
		Turkey	3
		TOTAL: 24 countries	125
Oceania	Australasia:	Australia	5
	Melanesia:	Solomon Islands	1

	Micronesia:<br><br><br><br>Polynesia:	Kiribati<br>Marshall Islands<br>Nauru<br><br>American Samoa<br>Cook Islands<br><br>TOTAL: 7	3<br>1<br>1<br><br>1<br>1<br><br>13
Europe	Eastern Europe:<br><br><br><br>Southern Europe:	Bulgaria<br>Romania<br>Ukraine<br><br>Albania<br>Bosnia and Herzegovina<br>Croatia<br>Greece<br>Portugal<br>Serbia<br><br>TOTAL: 9	3<br>6<br>1<br><br>2<br>6<br>1<br>1<br>1<br>1<br><br>22
		**89** countries	

1.3 Roles

Role	Number	%
Vet	68	62
Vet nurse/technician	11	10
Programme manager (non-veterinary)	24	22
Government worker (non-veterinary)	2	2
Other	5	5

Other:

- Communications officer for NGO
- Public health advisor
- Shelter volunteer x 2
- Volunteer with NGO

“Programme manager” also included founders and directors of NGOs working in canine sterilisation programmes.

Many respondents had multiple roles e.g. veterinary professional and programme manager, or veterinarian and government worker (e.g. veterinarian and head of rabies control and elimination programme, employee of Ministry of Agriculture, Natural Resources, Livestock and Fisheries and another vet was state coordinator of a prevention of rabies and other zoonoses program, state veterinarian, public health veterinarian or positions within Ministry of Agriculture).

Some of the respondents also worked in some aspect of academia or research, however their responses were included if they also had direct experience of working on sterilisation programmes with FRDs.

1.4 Organisations

Organisation	Number	%
NGO	82	75
IGO	3	3
Government (national, state)	20	18
Academic/research institute	3	3
Other	2	2

Other = independent consultants

1.5 Main aims

Aim	Number	%
Animal Health	29	26
Human health/Public health	10	9
Public health (veterinary)	20	18
Animal welfare	43	39
Education/training	2	2
Community engagement	2	2
Other	4	4

Other:

- Cannot pick one – human health, veterinary public health and animal welfare
- Conservation
- Environmental conservation and sustainable development
- Municipal council services

1.6 Other aims

Aim	Number	%
Animal Health	92	84
Human health/Public health	46	42
Public health (veterinary)	75	68
Animal welfare	93	85
Education/training	78	71
Community engagement	70	64
Other	7	6

- Wildlife conservation
- Advocacy
- Animal control

2. Interim survey

2.1 Countries of residence

Region	Sub-region	Country of residence	Number	TOTAL
Africa	Northern Africa	Morocco	1	2

		Tunisia	1	
	Sub-Saharan Africa	Burundi<br>Madagascar<br>Swaziland<br>Malawi<br>Kenya<br>Zimbabwe<br>South Africa<br>Tanzania<br>Uganda<br>Liberia<br>Ethiopia<br>Guinea-bissau<br>Mozambique<br>Namibia<br>Nigeria	1<br>1<br>1<br>1<br>1<br>2<br>2<br>1<br>1<br>1<br>1<br>1<br>1<br>1<br>1	15
Americas	North America	Canada<br>USA	2<br>6	2
	Latin America and the Caribbean	Belize<br>Panama<br>Costa Rica<br>Ecuador<br>Haiti<br>Chile<br>Mexico<br>Guatemala<br>St Kitts and Nevis	1<br>1<br>2<br>1<br>1<br>1<br>1<br>1<br>1	10
Asia	Eastern Asia	Taiwan	1	1
	South-Eastern Asia	Indonesia<br>Malaysia<br>Philippines<br>Thailand	2<br>2<br>3<br>1	4
	Southern Asia	Afghanistan<br>India<br>Pakistan<br>Nepal<br>Bangladesh<br>Sri Lanka	2<br>11<br>4<br>6<br>2<br>1	6
	Western Asia	Oman<br>Kuwait	1<br>1	2
Europe		UK<br>Portugal<br>Germany<br>Ukraine<br>Bulgaria<br>Romania<br>Denmark<br>Bosnia<br>Italy	18<br>1<br>1<br>1<br>1<br>1<br>1<br>1<br>1	9
Oceania		Australia<br>Fiji<br>NZ	5<br>1<br>1	3

2.2 Countries worked

Region	Sub-region	Country	Number
Africa	Northern Africa	Egypt<br>Tunisia<br>Morocco	1<br>2<br>2
	Sub-Saharan Africa	Gambia<br>Madagascar<br>Swaziland<br>Malawi<br>Kenya<br>Zimbabwe<br>South Africa<br>Tanzania<br>Burundi<br>Uganda<br>Ghana<br>Ethiopia<br>Liberia<br>Namibia<br>Mozambique<br>Guinea-bissau<br>Nigeria<br>Mauritius<br><br>TOTAL (**20** countries)	1<br>1<br>1<br>3<br>3<br>2<br>4<br>3<br>1<br>2<br>2<br>2<br>1<br>1<br>1<br>1<br>1<br>1<br><br>36
Americas	North America	USA<br>Canada	2<br>4
	Latin America and the Caribbean	Nicaragua<br>Guatemala<br>Panama<br>Costa Rica<br>Haiti<br>Chile<br>Peru<br>Mexico<br>Dominica<br>Dominican republic<br>Colombia<br>Bahamas<br>Brazil<br>Belize<br>Ecuador<br>Guyana<br><br>TOTAL (**17** countries)	5<br>4<br>1<br>3<br>4<br>2<br>4<br>4<br>1<br>4<br>1<br>1<br>2<br>1<br>4<br>1<br><br>48
Asia	Eastern Asia:	China<br>?Taiwan	3<br>1

	South<br>Eastern Asia	Cambodia<br>Indonesia<br>Laos<br>Malaysia<br>Philippines<br>Thailand<br>Vietnam	4<br>8<br>2<br>2<br>5<br>16<br>2
	Southern<br>Asia	Pakistan<br>Nepal<br>Afghanistan<br>Bangladesh<br>India<br>Iran (Islamic republic of)<br>Sri Lanka	4<br>14<br>2<br>3<br>30<br>1<br><br>9
	Western Asia	Iraq<br>UAE<br>Jordan<br>Syria<br>Turkey<br><br>TOTAL (**21** countries)	1<br>1<br>2<br>1<br>1<br><br>112
Oceania	Australasia:<br><br>Melanesia:<br><br>Micronesia:<br><br>Polynesia:	Australia<br><br>Fiji<br><br>Kiribati<br><br>Tonga<br>Cook islands<br><br>TOTAL (**5** countries)	3<br><br>1<br><br>1<br><br>1<br>1<br><br>7
Europe	Eastern<br>Europe	Ukraine<br>Bulgaria<br>Romania<br>Russia	3<br>2<br>7<br>1
	Southern<br>Europe	Albania<br>Bosnia<br>Portugal<br>Italy<br>Greece<br>Spain<br>Cyprus<br><br>TOTAL (**11** countries)	1<br>7<br>1<br>1<br>4<br>1<br>1<br><br>**29**

2.3 Roles

**Role**	**Number**	**%**
Vet	55	51
Vet nurse/technician	8	7

PM	25	23
CAHW/AHT	5	5
Researcher	6	6
Other	8	7

Other:

- Founder/co-founder NGO x 3
- Volunteer
- Committee member for NGO
- Animal catcher/handler and transportation
- Director
- Assistant manager of NGO

2.4 Organisations

Organisation	Number	%
NGO	76	71
IGO	1	1
Govt	16	15
Academic/research	13	12
Other	1	1

Other:

- Private vet

2.5 Main aims

Aim	Number	%
Animal health	23	22
Human health	3	3
VPH	23	22
Animal welfare	47	44
Community engagement	1	1
Education/training	5	5
Other	5	5

Other:

- All of above but main charter is animal control
- Sustainability, community conservation and development
- One health education (training vets/vet students on OH issues spanning vet med, human health and conservation issues) (education/training)
- Improve public safety (in relation to animals) + wellbeing of the animals
- 2 x wildlife conservation

2.6 Other aims

Aim	Number	%
Animal health	82	76.6
Human health	41	38.3
VPH	74	69.2
Animal welfare	88	82.2
Community engagement	74	69.2

Education/training	82	76.6
Other	29	27.1

Other:

- Majority = wildlife conservation (?22)
- Dog shelter
- Human-animal bond

3. Delphi round 1

3.1 Countries of residence

Region	Sub-region	Country of residence	Number	TOTAL
Africa	Northern Africa	Morocco	1	1
	Sub-Saharan Africa	Burundi<br>Malawi<br>Tanzania<br>Uganda	1<br>1<br>1<br>1	4
Americas	North America	Canada<br>USA	1<br>4	5
	Latin America and the Caribbean	Panama<br>Costa Rica<br>Chile<br>Mexico<br>Guatemala<br>St Kitts and Nevis<br>Nicaragua	1<br>1<br>1<br>1<br>1<br>1<br>1	7
Asia	South-Eastern Asia	Indonesia<br>Thailand	1<br>1	2
	Southern Asia	Afghanistan<br>India<br>Nepal<br>Pakistan	1<br>7<br>2<br>1	11
	Western Asia	Kuwait	1	1
Europe		UK<br>Portugal<br>Bosnia<br>Italy	5<br>1<br>1<br>1	8
Oceania		Australia<br>NZ	4<br>1	5

3.2 Countries worked

Region	Sub-region	Country	Number
Africa	Northern Africa	Tunisia<br>Morocco<br><br><br>Ethiopia<br>Malawi	1<br>2<br><br><br>1<br>2

	Sub-Saharan Africa	Kenya	1
		South Africa	2
		Tanzania	3
		Burundi	1
		Uganda	2
		Ghana	1
		Mauritius	1
		TOTAL (**11** countries)	17
Americas	North America	Canada	3
	Latin America and the Caribbean	Nicaragua	3
		Guatemala	2
		Panama	1
		Costa Rica	3
		Haiti	3
		Chile	2
		Peru	2
		Mexico	2
		Dominica	1
		Dominican republic	3
		Colombia	2
		Bahamas	1
		Brazil	2
		Belize	1
		Ecuador	3
		Guyana	1
		Paraguay	2
		Honduras	2
		TOTAL (**19** countries)	39
Asia	Eastern Asia:	China	2
	South Eastern Asia	Cambodia	3
		Indonesia	3
		Laos	2
		Thailand	3
		Vietnam	1
	Southern Asia	Pakistan	1
		Nepal	6
		Afghanistan	1
		India	12
		Iran (Islamic republic of)	1
		Sri Lanka	4
	Western Asia	Iraq	1
		UAE	1
		Jordan	1
		Syria	1
		Turkey	1
		TOTAL (**17** countries)	44

Oceania	Australasia:<br><br>Micronesia:<br><br>Polynesia:	Australia<br><br>Kiribati<br><br>Tonga<br><br>TOTAL (**3** countries)	3<br><br>1<br><br>1<br><br><br>5
Europe	Eastern Europe	Ukraine<br>Romania<br>Russia	1<br>4<br>1
	Southern Europe	Albania<br>Bosnia<br>Portugal<br>Italy<br>Greece<br>Cyprus<br><br>TOTAL (**9** countries)	1<br>4<br>1<br>1<br>1<br>1<br><br>15

3.3 Roles

Role	Number	%
Vet	17	39
Vet nurse/technician	5	11
PM	14	32
CAHW/AHT	0	-
Researcher	7	16
Other	1	2

3.4 Organisations

Organisation	Number	%
NGO	35	80
IGO	-	-
Govt	2	5
Academic/research	6	14
Other	1	2

3.5 Main aims

Aim	Number	%
Animal health	9	21
Human health	1	2
VPH	8	18
Animal welfare	22	50
Community engagement	-	-
Education/training	2	5
Other	2	5

3.6 All aims

Aim	Number	%
Animal health	30	6
Human health	16	36
VPH	29	66
Animal welfare	36	82
Community engagement	31	71
Education/training	34	77
Other	10	23

3.7 Rabies endemic

Yes	No
37 (84.1%)	7 (15.9%)

4. Delphi round 2

4.1 Countries of residence

Region	Sub-region	Country of residence	Number	TOTAL
Africa	Sub-Saharan Africa	Burundi<br>Tanzania	1<br>1	2
Americas	North America	Canada<br>USA	1<br>3	4
	Latin America and the Caribbean	Panama<br>Costa Rica<br>Chile<br>Mexico<br>Guatemala<br>St Kitts and Nevis	1<br>1<br>1<br>1<br>1<br>1	6
Asia	South-Eastern Asia	Indonesia<br>Thailand	1<br>1	2
	Southern Asia	Afghanistan<br>India<br>Nepal<br>Pakistan	1<br>6<br>2<br>1	10
	Western Asia	Kuwait	1	1
Europe		UK<br>Portugal<br>Bosnia<br>Italy	5<br>1<br>1<br>1	8
Oceania		Australia	3	3

4.2 Countries worked

Region	Sub-region	Country	Number
Africa	Northern Africa	Tunisia<br>Morocco	1<br>1
	Sub-Saharan Africa	Ethiopia<br>Malawi<br>Kenya<br>South Africa<br>Tanzania<br>Burundi<br>Mauritius	1<br>1<br>1<br>2<br>3<br>1<br>1
		TOTAL (**11** countries)	12
Americas	North America	Canada	3
	Latin America and the Caribbean	Nicaragua<br>Guatemala<br>Panama<br>Costa Rica<br>Haiti<br>Chile<br>Peru<br>Mexico<br>Dominica<br>Dominican republic<br>Colombia<br>Bahamas<br>Brazil<br>Belize<br>Ecuador<br>Guyana<br>Paraguay<br>Honduras	2<br>2<br>1<br>3<br>3<br>2<br>2<br>2<br>1<br>3<br>2<br>1<br>2<br>1<br>3<br>1<br>2<br>2
		TOTAL (**19** countries)	39
Asia	Eastern Asia:	China	1
	South Eastern Asia	Cambodia<br>Indonesia<br>Laos<br>Thailand<br>Vietnam	3<br>3<br>2<br>3<br>1
	Southern Asia	Pakistan<br>Nepal<br>Afghanistan<br>India<br>Iran (Islamic republic of)<br>Sri Lanka	1<br>6<br>1<br>10<br>1<br>4
	Western Asia	Iraq	1

		UAE<br>Jordan<br>Syria<br>Turkey<br><br>TOTAL (**17** countries)	1<br>1<br>1<br>1<br><br>42
Oceania	Australasia:<br><br>Micronesia:	Australia<br><br>Kiribati<br><br><br>TOTAL (**2** countries)	2<br><br>1<br><br><br>3
Europe	Eastern Europe	Ukraine<br>Romania	1<br>4
	Southern Europe	Albania<br>Bosnia<br>Portugal<br>Italy<br>Greece<br>Cyprus<br><br>TOTAL (**9** countries)	1<br>4<br>1<br>1<br>1<br>1<br><br>15

4.3 Roles

**Role**	**Number**	**%**
Vet	13	36
Vet nurse/technician	4	11
PM	12	33
CAHW/AHT	0	-
Researcher	6	17
Other	1	3

4.4 Organisations

**Organisation**	**Number**	**%**
NGO	28	78
IGO	-	-
Govt	2	6
Academic/research	5	14
Other	1	3

4.5 Main aims

**Aim**	**Number**	**%**
Animal health	7	19
Human health	1	3

VPH	6	17
Animal welfare	18	50
Community engagement	-	-
Education/training	2	6
Other	2	6

4.6 Rabies endemic

**Yes**	**No**
31 (86%)	5 (14%)
