# Supplementary material for: Priorities for Research into the Impact of Canine Surgical Sterilisation Programmes for Free-Roaming Dogs: An International Priority Setting Partnership
Source: Animals (Basel). 2021 Jul 30;11(8):2250. doi: 10.3390/ani11082250 (PMC8388514; doi:10.3390/ani11082250)
Supplement: Supplementary file 1 [file animals-11-02250-s001.zip › File S4 Excluded questions submitted to the initial scoping survey.pdf]

File S4 Excluded Submissions from the initial scoping survey

Out of scope submissions (n=168)

Table 1: Qs submitted that were about carrying out canine surgical sterilisation programmes but were not related to impact (n= 69)

Question type	No.	Submitted questions
Ethics	2	• Why have animal organizations not opposed early age neutering for homeless dogs?<br>• I can understand why it is not practically possible to sterilise all dogs caught for vaccination on a rabies vaccine drive, but is it ethical to only vaccinate them and not sterilise them? It can be argued that vaccinating them better than doing nothing and protecting public health as well as the dogs
Other treatments at time of surgery	4	• Will using nexguard or other tick-demodex medicines at the time of sterilization have a negative effect on the outcome of the parastie medication?<br>• Is it significantly beneficial to vaccinate them for distemper/parvo at the time of sterilization?<br>• Would you also simultaneously carry out vaccination for infectious diseases such as distemper, leptospirosis?<br>• What's the cost/benefit of distemper/parvo vaccination of free ranging dogs in an ABC program?
Education components	9	• Are the owners/caretakers of the "owned" or "partially -owned" dogs provided information on bite awareness and zoonotic diseases (signs of symptoms of hookworm infection in human etc)?<br>• What information is provided to educate the public on canine rabies, bite awareness, and bite reporting?<br>• How do we ensure that any educational component of a project has measuring impact rather than just numbers reached?<br>• How do you educate the community and what to you educate the community on in regards to roaming dogs?<br>• Does TRN with no human interaction have any benefit or should it always include some human education?<br>• Are there some communities who are more accepting of rabies vaccination and sterilisation programmes than others? If so, does this correlate with the provision of education programmes?<br>• Do local community members actively help by searching for and/or bringing street dogs for rabies vaccination and sterilisation? If so, does this correlate with the provision of education programmes?<br>• What is the impact of just sterilizing vs sterilizing and educating? What is the difference of just educating without sterilizing? (Would people supervise their pet

		more and not allow it to roam and mate?... if this is the case than potentially sterilization is not needed.<br>• Can the sterilization program alone without education and sensitization of the community enough to have an impact on total free-roaming dog population?
Procedure	10	• Is using an anethesia machine much better when running mobile spay neuter programs then injectible drugs without the anethesia machine?<br>• If a dog is not healthy, has body type of 1-2 for example, skinny and obvious parastites, internal or external, is it still OK to do surgery if there may not be another chance?<br>• What age do you recommend sterilisation (we do it as early as 3 months)<br>• How long should street dogs be kept/housed before released?<br>• What type of care should they have during that period?<br>• How do you control Parvo/Distemper outbreaks during a mass sterilization campaign when sterilizing a general population of unvaccinated street puppies?<br>• Is there significant difference between surgical outcomes of flank vrs midline spays?<br>• How long should dogs be kept post op before releasing back on the streets?<br>• What quarenteen systems need to be implemented?<br>• What is or where are the general guidelines and Operational Plan for such programs; are all vets fully qualified? are student vets supervised? how long do programs go for? policies on euthanasia?
Dog catching	2	• What is the most humane practical way of catching street dogs?<br>• How will these dogs be captured for the intervention?
Miscellaneous	4	• What is the most important aspect of a sterilisation program?<br>• How does sterilization helps in prevent zoophytic diseases?<br>• Does the sterilization surgery have any impact on parvo?<br>• Shed less zoonotic parasites?
Consent	2	• What do you do if you sterilize an owned free roaming dog and the owner objects?<br>• Are consent forms necessary?
How information is shared/disseminated	2	• How (if at all) are the above statistics gathered and disseminated region/country/world-wide?<br>• Which data tracking recording software is available?
Post-surgery logistics/issues	4	• Who will look after the welfare of these dogs?<br>• after the sterilisation program, who will mange the dogs once it is released back to its community? will it have to fend itself on the streets and be a public menance to the local comunity?

		• What is considered good post-work practices after a sterilization program?<br>• How do you manage surgical sites/infections/pain relief post operatively?
Shelter management	1	• What are the basics when planning a shelter?
Qs specific to own programme	2	• Is an animal census part of the program?<br>• What is the dog/cat population in the communities where I work?
Alternatives to surgery – availability, feasibility, comparisons, costs	7	• Comparison of different methods (chemical vs regular castration) on time, impact, complications.<br>• Are there any new sterilization techniques being developed that don't require surgery?<br>• Why is there not more research into safe injectable or oral long acting birth control in roaming female dogs?<br>• The best method for sterilisation - Drugs or surgical intervention?<br>• Is chemical castration a feasible option for sterilizing male dogs? Can it be used as an alternative to neutering when administering animal birth control on a mass scale?<br>• Are cheaper methodology to spay and neuter in the pipeline?<br>• What benefits does surgical sterilisation offer over injectable in relation to all of these aspects?
Programme management but not linked to impact	20	• Is the program led by the local ministry of agriculture/public health or an NGO?<br>• How do we ensure that an ABC project has a robust strategy that includes pre-project assessment and post project monitoring and evaluation?<br>• What measures should a country take to effectively maintain and sustain sterilization method as a tool for dog population control?<br>• How do you ensure that additional factors that may affect the impact of a rabies vaccination program (such as the dog meat trade) are considered by authorities when advocating such a program?<br>• Is there a local canine rabies control program in conjunction with the animal population control program?<br>• Multi collaborative initiatives, what are the common goals and how can they be achieved quicker by working together?<br>• How do we build in greater sustainability into projects so that local partners stakeholders are in as strong a position as possible to continue to manage and control the problem effectively?<br>• Stakeholders to involve in such an approach<br>• Stray dogs have owners. What legal consideration will one put in place to ensure owners compliance<br>• How can we ensure that ABC projects don't produce an over reliance on whoever is implementing that project?<br>• Should rabies control be prioritised over dog population control?

		• Where in the government should be placed sterilization programs?<br>• What can local government councils do to make such programs more cost effective?<br>• What steps can federal, state, and local government take to help communities develop to a point where there is no need for such programs, or have access to such services so it does not get to a point of mass sterilization?<br>• Is it better to work intensively in one smaller area or over a large area for population control?<br>• Is it better to work intensively in one smaller area or over a large area for health benefits to dogs?<br>• How best to define a boundary for a pilot sterilization programme?<br>• Should we even be recommending sterilization when rabies programmes are just initiating? Public health organizations say that sterilizing distracts from vaccinating, which should be the main focus when in a rabies endemic country. I agree with this, and when funds are limited I would say: vaccinate<br>• In mass vaccination campaigns where a day may be spent vaccinating a 10km radius, would it be beneficial to actually have sterilisations carried out at the same time or just to focus on vaccinations and return at a later date for sterilisation?<br>• So you ask owners for a financial contribution?

Table 2: Qs related to FRD populations but not related to impact of canine surgical sterilisation programmes (N= 35)

Question type	No	Example questions
Reproduction in FRD	4	• How many offspring can an unsterilized pair of dogs and their children produce in their lifetime?<br>• Why does the puppy breeding season seem to occur earlier each year in India?<br>• Why is the puppy breeding season during Winter in Rajasthan when temperatures are coldest?<br>• What are the population dynamics of the population?
Lifespan	3	• What is the likely life span of free roaming dogs?<br>• How long lives a dog<br>• What is the average lifespan for free-roaming dogs?
About vaccination only	16	• How essential is it to revaccinate dogs every year if rabies is not known to be endemic to a local region?<br>• In Mexico, we have vaccinated dogs and cats from dogs and cats from the first month of age against rabies for more than 25 years. This has allowed us to achieve the elimination of human rabies transmitted by dogs. Now that it is not an endemic disease which vaccination scheme would be appropriate?<br>• How many animals getting a first rabies vaccination get any more in their lifetime?

		• How many rabies vaccinated animals get more than ome vaccination in their life?<br>• How often should the rabies vaccine be repeated in free-roaming dog populations (especially rural residents)? Should it be every year? Every 2 years? Every 3?<br>• What is the minimum amount of dogs to vaccinate in a population to be considered "rabies free"?<br>• Effect of vaccinating underage animals vs not given circumstances of catch-release<br>• How young should you vaccinate with Rabies vaccines in countries where Rabies is endemic and it is unknown if you will have another chance to vaccinate these young dogs?<br>• Is the vaccine inexpensive enough and is it worth Rabies vaccinating before 12 weeks of age?<br>• How early can the rabies vaccine be safely administered to puppies?<br>• Do the risks outweigh the positives when administering rabies younger than 16 weeks?<br>• Are we giving 1 year vs 3 year vaccinations?<br>• What is the best method to ensure vaccinations are handled properly (correct temp, etc.) to give them the best chance of being efficacious?<br>• How can I calculate an adequate sample size to do a seroconversion study for the rabies vaccine?<br>• How to conduct a mass vaccination program (with or without sterilisation) in an area where free-roaming dogs are not tolerated?<br>• Our priority is animal health and we use rabies vaccination as a method of engaging with owners and community members who are generally more interested in human health. Are there any ways of demonstrating the effectiveness of vaccination campaigns to communities whose animals we treat?
Rabies surveillance	3	• Does surveillance help control rabies?<br>• What is the best method to track rabies disease prevalence in a population of free roaming dogs?<br>• How can we plan surveillance during the sterilization campaign?
Other components of DPM programmes	6	• It is important to tie in education and dog management programs with surgical sterilization for communities with large free-roaming dog populations. Is this study going to be looking at any of the other components such as a dog management program? Some key things to look at would be the structure of the program, engagement, implementation and challenges.<br>• Is education a part of the sterilization campaign?<br>• Impact of local training of lay people to vaccinate. Cost savings and effect<br>• What other services would the program offer whilst doing sterilization programs on communities with limited veterinary services?<br>• How much effort to put into other components of a program (education, vaccination, garbage control, etc) to have an impact on population size?

		• Is sterilization only enough to spread awareness about dog welfare, Rabies or it should be sold with shelters and emergency treatment services? - i.g - In India, its really difficult for us to motivate people to bring their dogs for sterilization if you don't provide other veterinary services. The term we use is community or semi-owned dogs, people feed them but they don't take full ownership
Attitudes to FRDs	1	• How does TNR work in an increasingly urban area where free-roaming dogs are not welcome and cleanliness are a priority?
Effects of ownership	2	• How much does responsible ownership (care, feeding, etc) affect the behaviours (ie.,roaming, aggression, mating) of FRD?<br>• How does animal husbandry enhance or impede the role of domestic dogs as reservoirs for pathogens or incidental hosts?

Table 3: Qs related to impact of canine surgical sterilisation programmes but not asking a specific question about impact on free-roaming dogs (n=23)

Question type	No	Example questions
Specific to own programme	1	• What impact does my program (spay/neuter) have on the dog/cat population?
Overall impact	5	• What are the longitudinal effects of these programmes?<br>• What will be the long term effect of sterilization in dogs?<br>• What can be done to make a sterilisation program successful?<br>• What are the long term prospects of sterilised dogs?<br>• What is the impact of the sterilization program - are we making a difference?
Ambiguous (covered by more specific questions)	7	• What effects does sterilization have on free-roaming dogs in terms of behavior changes?<br>• Dog behaviour?<br>• Are there any empirical evidence of improved health, docility or changed behaviour in sterilised free-roaming dogs?<br>• Does sterilisation affect dog behaviour<br>• Does the sterilization programs alone helps to prevent zoonosis?<br>• Do sterilised free roaming dogs still pose a danger to the human population?<br>• How does sterilization actually impact dog behavior; by breed, age and guardianship status
Misc	10	• Why there is no successful sustainable sterilization program which has achieved: Reduced population of street dogs, improved welfare of dogs, increased the sense of co-existence between humans and dogs, a decline in numbers of human-dog conflicts

		• What % and duration of sterilization needs to be in place before this is an expected guardianship behavior?<br>• Do such programmes aim to involve communities to alter their attitudes towards free-roaming dogs?<br>• What impact does a sterilization programme have on neighbouring communities where the programme is not active?<br>• Does sterilization of dogs have socioeconomic impact on the society?<br>• How do you stop the movement of unvaccinated dogs into an area already treated?<br>• Has it been indicated in any survey that high dog population is a high risk factor in rabies prevalence?<br>• Is there a difference in human attitudes toward dogs when veterinary services are offered for free versus owners having to pay for service?<br>• Do these programs help start long term/permanent local, organized dog shelter/adoption organizations?<br>• What effect does overuse of antibiotics in the place of good practice and surgical sterility have on the dog and human population in the future?

Table 4: Qs not about FRDs (n=4)

Question type	No	Example questions
Working dogs	2	• Is there an impact of sterilization on the performance of dogs kept for herding, guarding or working?<br>• Does sterilisation reduce dog guarding behaviour of property?
Confined dogs	2	• Relationship between sterilization and confined dog aggression?<br>• How much does neutering owned and non-roaming animals have an impact on the free-roaming population?

Table 5: Qs related to monitoring and evaluation of canine surgical sterilisation programmes but not linked to impact (n=37)

Question type	No	Example questions
Most effective method for identification of sterilised dogs	9	• What is the best method(s) to permanently ID a free roaming dog as sterilized?<br>• How to traceback these sterilised dogs should it is needed to vaccinate these dogs in the rabies controlled area?<br>• How best to keep track of sterilized and Rabies vaccinated dogs?<br>• How do you track/mark stray dogs that have been sterilised?<br>• What is the best way (or combination of ways) to mark sterilised street dogs? ie tattoos, ear notches etc

		• How can we track surgery and vacc? Eg set up a national database that all organisations would use.<br>• Is there a need to mark the sterilised and vaccinated dogs and what are the costs involved?<br>• What is the most effective way to tag vaccinated, neutered/spayed dogs? We use spray paints for vaccinated dogs but these come off in less than a week.<br>• How can the vaccinated dogs be easily identified to avoid recapture or disposal?
Most effective method for identification of vaccinated dogs, if vaccinating at additional times to sterilisation	11	• What is the best method to ID vaccinated animals?<br>• What is the best method to track vaccination rates in a population?<br>• Microchipping street dogs after rabies vaccination?<br>• How do you monitor booster vaccinations in the animals<br>• What is the best way to mark vaccinated dogs?<br>• How should the re-vaccination year be marked?<br>• Is there any tracking software/data record keeping software that enables us to identify dogs again when it comes to re-vaccination and update their same records?<br>• how do you know when have they been vaccinated?<br>• how can I find out that next year they are the same population?<br>• How is the best way to record rabies vaccination status in the short and long term?<br>• We mark the ears of the dogs who has been vaccinated and sterilized (ear notch). How can I differentiate between a dog who has been vaccinated a year ago vs a few months ago? Apart from community members having that information, and maybe knowing that dogs recently vaccinated are from a different location, how can we effectively know the difference?
Methods for monitoring welfare, behaviour and human KAP impacts	3	• How do we measure welfare in free-roaming dog populations?<br>• How can we better measure the behaviours of dogs and an indicator to community behaviours towards the dogs?<br>• How can we produce better measures that directly correlate the impact of a ABC project with changes in community attitudes and behaviours?
Most effective method to measure dog population in an area	9	• How do we measure the size of dog populations?<br>• Are population studies (dog census) conducted as part of the sterilization program?<br>• How is a free-roaming canine population size assessed?<br>• How would you measure or estimate the number of dogs, if someone is going to perform mass dog sterilization in any specified area?<br>• How can I calculate the population of animals that exist in a locality?<br>• How will one determine the population of the stray dogs to estimate numbers for sterilisation

		• How do we conduct an effective and useful dog survey?<br>• What is the most effective way to count dogs in an area?<br>• How often should street dog population surveys be done?
How can the relative impacts of sterilisation and vaccination be determined in a combined programme	4	• Can the relative impacts of sterilisation and vaccination be determined?<br>• Are there any models comparing vaccination with and without neutering?<br>• How do you determine and promote the importance of sterilisation in relation to a rabies vaccination program?<br>• How do you measure the changes of attitudes and behaviours towards dogs in relation to rabies vaccination, sterilisation and both together?
Most effective method to determine vaccination coverage in sterilised dogs	1	• How to calculate the rabies vaccination coverage among these sterilised dogs in order to ensure that these dogs is protected against rabies?

Known Unknowns (n=18)

Questions that could be answered by evidence review

1. **Is sterilisation necessary/effective or can vaccination alone be used to control rabies successfully? (13)**

- Can only sterilization and vaccination controls rabies? Can only vaccination without Birth Control program is possible for Controlling rabies?
- Is it even necessary or is yearly vaccination of every available dog the best option practically and epidemiologically?
- Do ABC or surgical sterilization proved effective as compared to Mass Dog Vaccination in controlling rabies?
- How do you rate effectiveness of sterilisation programmes vi-a-vis mass immunisation against rabies for control of dog-bite related rabies?
- What is the impact of vaccinating and not sterilizing a part of the population on the rabies transmission?
- What is the impact on Rabies control?
- Whether CNR is a needed component of rabies elimination and how it can help?
- Do ABC or surgical sterilization proved effective as compared to Mass Dog Vaccination in controlling rabies?
- Can you eradicate canine Rabies without sterilization of roaming dogs?
- Does surgical sterilisation impact on rabies transmission?
- How does sterilization helps in prevent rabies?
- Sterilizing itself does prevent rabies?
- Can sterilisation programme really help reduce canine rabies ?

2. **What indicators can be used to measure the impact of a sterilisation programme? (5)**

- What are the best indicators of change to use to monitor the success of a CNR programme in terms of population reduction?

- What are the most useful measurements of the impact of a sterilisation programme? e.g. overall population numbers/individual animal health/number of lactating females/number of entire male dogs/something else?
- How is the success of a sterilisation program measured?
- Which methods for monitoring would be effective for a CNVR campaign?
- How are impacts of such sterilisation programmes evaluated?
