# Supplementary material for: Priorities for Research into the Impact of Canine Surgical Sterilisation Programmes for Free-Roaming Dogs: An International Priority Setting Partnership
Source: Animals (Basel). 2021 Jul 30;11(8):2250. doi: 10.3390/ani11082250 (PMC8388514; doi:10.3390/ani11082250)
Supplement: Supplementary file 1 [file animals-11-02250-s001.zip › File S5 Questions for final prioritisation.pdf]

Impact of Canine Surgical Sterilisation Programmes Priority Setting Partnership

File S5 Short list of CIRQs for final prioritisation.

INDIVIDUAL RANKING OF QUESTIONS/ UNCERTAINTIES

Please spend some time reviewing these uncertainties about the impact of canine surgical sterilisation programmes. In the online survey you will be asked to rank them from 1–26, **1 being the most important**, and **26 being the least important**, in your opinion, for future research to address. You may find this easier to complete on this document prior to completing the online survey.
Make a note of any comments you may have in the far right hand column.

ID	Uncertainty	Your ranking (1-26)	Notes
A	In order to affect the size of a dog population, what proportion of the population needs to be sterilised and over what time period?		
B	How does dog population turnover change in response to a sterilisation programme?		
C	What effect does targeting of female dogs only have on the impact of sterilisation programmes?		
D	Does sterilisation affect the rate of dog-bites in people?		
E	Do sterilisation programmes affect community behaviour (human-dog interactions) towards free-roaming dogs?		
F	Do sterilisation programmes affect community perception of free-roaming dogs?		
G	Do areas with sterilisation and vaccination programmes have a lower incidence of rabies in dogs and humans than areas with vaccination only programmes (that achieve the same level of vaccination coverage)?		
H	Does age at sterilisation have any effect on subsequent changes in population size, behaviour, longevity and health?		
I	Are sterilisation programmes cost-effective taking into account all programme objectives?		
J	What factors regulate dog population size, both natural and human mediated?		
K	What are the effects of one-off or intermittent sterilisation (with or without vaccination) programmes in comparison to consistent programmes in an area? What frequency is optimal?		

Impact of Canine Surgical Sterilisation Programmes Priority Setting Partnership

L	How common is training of local veterinary personnel a component of sterilisation programmes. When implemented, is there a measurable improvement in local veterinary care?		
M	How do sterilisation programmes (of different sizes and durations) affect the size of dog populations?		
N	Is sterilisation associated with a change in expected lifespan?		
O	How do sterilisation programmes affect birth rates in the population?		
P	Does sterilisation have any direct effects on rabies transmission e.g. in terms of behavioural changes?		
Q	Are changes in policy e.g. ending of culling or government funding for dog population management, one impact that may be seen after a sterilisation programme?		
R	What are any negative effects on free-roaming dogs as a result of sterilisation programmes e.g. surgical complications, welfare implications?		
S	What logistical factors affect community engagement/participation in sterilisation programmes?		
T	What are the most common causes of death for free-roaming dogs and are they different in sterilised dogs?		
U	Does sterilisation affect free-roaming dog interactions with humans?		
V	Does sterilisation affect the incidence of dog-to-dog aggression?		
W	Does sterilisation affect rates/risk of specific health conditions (e.g. skin conditions, road traffic accidents)		
X	Are sterilisation programmes cost-effective if rabies control is the only objective?		
Y	Is sterilisation associated with a change in body condition score?		
Z	Do sterilisation programmes result in more free-roaming dogs being adopted or classed as being ‘owned’ in an area?		
