# Supplementary material for: Priorities for Research into the Impact of Canine Surgical Sterilisation Programmes for Free-Roaming Dogs: An International Priority Setting Partnership
Source: Animals (Basel). 2021 Jul 30;11(8):2250. doi: 10.3390/ani11082250 (PMC8388514; doi:10.3390/ani11082250)
Supplement: Supplementary file 1 [file animals-11-02250-s001.zip › Table S1 Reprise Reporting Guidelines.pdf]

# Table S1 REPRISE Reporting Guidelines

| No | Item | Descriptor and/or *examples* |
|---|---|---|
| **A** | **Context and scope** | |
| 1 | Define geographical scope | Global |
| 2 | Define health area, field, focus | Canine surgical sterilisation programmes for free-roaming dogs, identifying research priorities |
| 3 | Define end-users of research | People working on or involved in any programme involving canine surgical sterilisation for free-roaming dogs |
| 4 | Define the target audience of the priorities | Policymakers, funders, researchers |
| 5 | Identify the broad research area | One health, Public health, Veterinary research, DPM research, Animal welfare research |
| 6 | Identify the type of research question | Intervention, evaluation, implementation |
| 7 | Define the time frame | Medium and long-term priorities |
| **B** | **Governance and team** | |
| 8 | Describe selection of the leadership and management team | Materials and methods, lines 102-107 |
| 9 | Describe the characteristics of the team, and the networks they represent | Materials and methods, lines 102-107 |
| 10 | Describe any training or experience in priority setting | Materials and methods, lines 107-108<br>The first author also attended a JLA PSP final priority setting workshop as an observer in June 2018 |
| **C** | **Framework for priority setting** | |
| 11 | State the framework used (if any) | James Lind Alliance |
| **D** | **Stakeholders or participants** | |
| 12 | Define the inclusion criteria for stakeholders involved in priority-setting | non-governmental organisations, government, research institutes with experience of field work with free-roaming dogs and sterilisation programmes, policymakers |
| 13 | State the strategy or method for identifying and engaging stakeholders | Recruitment by email through existing professional and personal networks of steering group, Dogs Trust Worldwide and Global Alliance for Rabies Control; Snowballing; Social media |
| 14 | Indicate the number of participants and/or organisations involved | Results, |
| 15 | Describe the characteristics of stakeholders | Stakeholder group, demographic characteristics, areas of interest and expertise, discipline, affiliations |
| 16 | State if reimbursement for participation was provided | No reimbursement or financial incentive for participation |
| **E** | **Identification and collection of research priorities** | |
| 17 | Describe methods for collecting priorities from stakeholders | Online questionnaire |
| 18 | Describe methods for collating and categorizing priorities | Thematic anaysis |
| 19 | Describe methods and reasons for removing priorities | Based on scope and clarity |
| 20 | Describe methods for refining or translating priorities into research topics or questions | Reviewed by authors and verified by steering group |
| 21 | Describe methods for checking whether research questions or topics have been answered | Evidence search for systematic reviews or published guidelines |
| 22 | Describe number of research questions or topics | Results, Figure 2 |
| **F** | **Prioritisation of research topics/questions** | |
| 23 | Describe methods and criteria for prioritising research topics or questions | Online interim prioritisation survey and Delphi survey |

| | | |
|---|---|---|
| 24 | Provide reasons for excluding research topics/questions | Lowest ranking scores and proportion of participants who agreed that it was a top 10 priority |
| **G** | **Output** | |
| 25 | Specificity of research priorities are clear | Results, Table 3 |
| **H** | **Evaluation and feedback** | |
| 26 | Describe how the process of prioritization was evaluated | Free- text box for comments |
| 27 | Describe the approach for feeding back priorities to stakeholders and/or to the public; and how feedback was addressed and integrated | Lay summary of results sent to all participants, presentation of results at various conferences, paper to be submitted for peer-reviewed publication |
| **I** | **Implementation** | |
| 28 | Outline the strategy or action plans for implementing priorities | To be completed |
| 29 | Describe evaluation of impact | To be completed |
| **J** | **Funding and conflict of interest** | |
| 30 | State sources of funding | Dogs Trust Canine Welfare Grant and University of Nottingham School of Veterinary Medicine and Science |
| 31 | Outline the budget and/or cost | n/a – part of PhD project |
| 32 | Provide declaration of conflict of interest | No conflict of interest |
