# Supplementary material for: Priorities for Research into the Impact of Canine Surgical Sterilisation Programmes for Free-Roaming Dogs: An International Priority Setting Partnership
Source: Animals (Basel). 2021 Jul 30;11(8):2250. doi: 10.3390/ani11082250 (PMC8388514; doi:10.3390/ani11082250)
Supplement: Supplementary file 1 [file animals-11-02250-s001.zip › Table S2 Evidence search.pdf]

Table S2 Details of search terms used and databases searched in evidence search

Databases searched:	CAB Abstracts (1910-present)<br><br>Medline In-Process and Non-Indexed Citations and Ovid Medline (1946-present)	Via University of Nottingham library
Search terms:	dog.mp. OR dogs.mp. OR canine.mp. OR canines.mp. OR canis.mp. OR exp Dogs/ AND sterilis$.mp. OR steriliz$.mp. OR dog population management.mp. OR animal birth control.mp. OR neuter$.mp. OR spay$.mp. OR spey$.mp. OR castrat$.mp. OR ovariohysterectomy.mp. OR fertility control.mp. OR gonadectomy.mp. OR ABC.mp	
Date searches performed:	06/09/2019	
