# Supplementary material for: Priorities for Research into the Impact of Canine Surgical Sterilisation Programmes for Free-Roaming Dogs: An International Priority Setting Partnership
Source: Animals (Basel). 2021 Jul 30;11(8):2250. doi: 10.3390/ani11082250 (PMC8388514; doi:10.3390/ani11082250)
Supplement: Supplementary file 1 [file animals-11-02250-s001.zip › Table S3 Questions for interim prioritisation.pdf]

Table S3 Long list of CIRQs for interim prioritisation and number of submitted questions contributing to each CIRQ

**Theme**		**Question**	**No. submitted Qs contributing to Q**
**Dog Behaviour**			
	1	Does sterilisation affect free-roaming dog interactions with humans?	14
	2	Does sterilisation affect the rate of dog-bites in people?	22
	3	Does sterilisation reduce the incidence of dog-to-dog aggression?	8
	4	Does sterilisation affect the roaming behaviour of dogs, in terms of distance/area?	9
	5	Does sterilisation change contact rates between free-roaming dogs?	1
	6	Are there any behavioural changes in unsterilised males where programmes focus on sterilisation of females?	2
	7	What is the social structure of free-roaming dog populations and how does surgical sterilisation affect this social structure?	11
**Health and welfare**			
	8	Is sterilisation associated with a change in expected lifespan?	31
	9	What are the most common causes of death for free-roaming dogs and are they different in sterilised dogs?	2
	10	Does sterilisation affect rates/risk of specific health conditions (e.g. skin conditions, road traffic accidents)	12
	11	What is the impact of sterilisation programmes on incidence of TVT?	4
	12	Is sterilisation associated with a change in body condition score?	3
	13	Are any changes in welfare seen in the whole free-roaming dog population, or only in sterilised dogs?	10

**Human behaviour change**			
	14	What sociocultural or religious barriers may affect participation in sterilisation programmes in different settings?	8
	15	What logistical factors affect community engagement/participation in sterilisation programmes?	15
	16	Do sterilisation programmes affect community perception of free-roaming dogs?	17
	17	Do sterilisation programmes affect community behaviour (human-dog interactions) towards free-roaming dogs?	12
	18	Do sterilisation programmes result in more free-roaming dogs being adopted or classed as being 'owned' in an area?	8
**Population size and structure**			
	19	What factors regulate dog population size, both natural and human mediated?	15
	20	How do sterilisation programmes (of different sizes and durations) affect the size of dog populations?	23
	21	In order to affect the size of a dog population, what proportion of the population needs to be sterilised and over what time period?	42
	22	How can we establish what the dog population turnover is (in terms of birth rates, age and sex specific death rates and in-out migration rates) and therefore monitor how it changes in response to a sterilisation programme?	8
	23	Do sterilisation programmes affect birth rates in the population?	9
	24	Do sterilisation programmes affect age and sex specific death rates in the population?	4
	25	Do sterilisation programmes affect migration rates in a population?	6
	26	Do sterilisation programmes affect the age structure of the dog population?	3
**Logistics**			

	27	Does age at sterilisation have any effect on subsequent changes in population size, behaviour, longevity and health?	7
	28	What effect does targeting of female dogs only have on the impact of sterilisation programmes?	18
	29	What are the effects of one-off or intermittent sterilisation (with or without rabies vaccination) programmes? What frequency is optimal?	9
	30	Do inaccessible dogs have an effect on the impact of the programme?	4
	31	How common is training of local veterinary personnel a component of sterilisation programmes. When implemented, is there a measurable improvement in local veterinary care?	4
	32	Are there any differences in impact between sterilisation programmes conducted in urban and rural areas?	2
**Policy and economics**			
	33	Do the outcomes of sterilisation programmes match stakeholder expectations?	1
	34	Are changes in policy e.g. ending of culling or government funding for dog population management, one impact that may be seen after a sterilisation programme?	4
	35	Are sterilisation programmes cost-effective taking into account all programme objectives?	9
	36	Are sterilisation programmes cost-effective if rabies control is the only objective?	13
**Other animal populations**			
	37	Do canine sterilisation programmes have an impact on other domestic animal populations? e.g. incidence of attacks on livestock, free-roaming cat populations	2
	38	Do canine sterilisation programmes have an impact on wildlife populations?	2
**Negative impacts**			
	39	What are any negative effects on free-roaming dogs as a result of sterilisation programmes e.g. surgical complications, welfare implications?	17
**Rabies control**			

	40	Is sterilisation or rabies vaccination more of an incentive for community participation in programmes?	5
	41	Does sterilisation affect the rate of rabies vaccination coverage decline? (If so, what is optimal in terms of sterilisation and vaccination coverage and time between campaigns?)	11
	42	How often should sterilised dogs be re-vaccinated against rabies?	36
	43	Do areas with sterilisation and vaccination programmes have a lower incidence of rabies in dogs and humans than areas with vaccination only programmes (that achieve the same level of vaccination coverage)?	14
	44	In combined programmes, to what extent are changes in perception and attitudes/behaviour towards free-roaming dogs due to the sterilisation or vaccination component?	5
	45	Does sterilisation have any direct effects on rabies transmission e.g. in terms of behavioural changes?	3
	46	Are owners/carers of free-roaming dogs more likely to bring sterilised dogs to be vaccinated against rabies than unsterilised dogs?	11
	47	Does performing the sterilisation surgery at the same time as the vaccination have any effects on immunity?	10
